# Peripapillary structural and microvascular alterations in early dysthyroid optic neuropathy

**DOI:** 10.1186/s40662-022-00301-6

**Published:** 2022-08-09

**Authors:** Yufei Wu, Qiaoli Yang, Liujun Ding, Yunhai Tu, Xiaoyu Deng, Yan Yang, Meixiao Shen, Qinkang Lu, Fan Lu, Qi Chen

**Affiliations:** 1 The Affiliated People’s Hospital of Ningbo University, Ningbo, Zhejiang China; 2 School of Ophthalmology and Optometry, Wenzhou Medical University, 270 Xueyuan Road, Wenzhou, 325027 Zhejiang China

**Keywords:** Retina, Microvasculature, Density, Thickness, Optical coherence tomography angiography, Thyroid-associated ophthalmopathy

## Abstract

**Background:**

To explore the changes in blood supply and structure around the optic nerve head (ONH) in thyroid-associated ophthalmopathy (TAO) patients with suspected dysthyroid optic neuropathy (DON).

**Methods:**

TAO patients [19 with DON; 24 non-DON (NDON); 20 with equivocal DON (EDON)], and 34 control subjects were examined. Optical coherence tomography angiography (OCTA) was used to obtain peripapillary retinal nerve fiber layer (p-RNFL) and vessel density parameters, including the ONH whole image vessel density (ONH-wiVD) and the radial peripapillary capillary vessel density (RPC-VD) in early DON.

**Results:**

Although there were no differences in p-RNFL thickness among the groups, there were differences in the ONH-wiVD of each grid section and the RPC-VD in all areas (*P* < 0.01). Compared with healthy controls, the EDON eyes had significantly lower RPC-VDs in all aeras (*P* < 0.05).The peripapillary region was further divided into eight sectors, and the RPC-VD in the temporal upper, superior temporal, and temporal lower sectors in the EDON group were significantly lower than in the controls. The visual impairment was closely related to the loss of peripapillary capillary vessel density. Univariate correlation analysis showed that the ONH-wiVD and RPC-VD of the TAO groups were negatively correlated with the intraocular pressure (r = − 0.296, *P* = 0.006; r = − 0.258, *P* = 0.016 respectively).

**Conclusions:**

EDON patients had significantly lower ONH-wiVD and RPC-VD than control subjects, and the temporal and upper VDs were more likely to be affected in the early stage of TAO. The combined use of spectral domain optical coherence tomography and OCTA technologies offer a new method for early diagnosis of suspected DON patients.

## Background

Thyroid-associated ophthalmopathy (TAO) is an autoimmune disorder that predominantly affects patients with thyroid dysfunction and causes swelling and inflammation in the muscles and tissue surrounding the eye [1, 2]. According to the affected eye tissue, it has a series of clinical manifestations ranging from eyelid contraction and protrusion of the eyeball to corneal exposure and compression of the optic nerve [3]. Approximately 4–8% of TAO patients have severe sight-threatening pathology due to dysthyroid optic neuropathy (DON) [4, 5], which is the most serious ocular complications associated with TAO. The exact pathogenesis of DON is uncertain, but it may be related to mechanical compression of extraocular muscles, blood flow changes, and inflammation [6]. Recent studies have found that the compression of the rectus muscle may cause optic nerve ischemia or axon reduction, and the associated blood vessel changes may be related to the development of DON [7, 8]. The clinical diagnosis of DON is based mainly on visual function defects. However, it is sometimes difficult to identify optic nerve and retinal tissue involvement by functional tests because they are subject to change and the findings are not necessarily congruent [9]. Therefore, identifying early objective indicators of DON in TAO, even before the clinical diagnosis of DON, is important for identifying new therapeutic targets in the prevention of advanced disease.

Decreased retinal blood flow is one of the pathological characteristics of DON [10]; however, the classification of DON severity is still based on daily practice and subjective clinical visual function tests. Because of diagnostic technical limitations, there are no objective indicators of the onset, progression, and severity of DON. More recently, optical coherence tomography (OCT) has become widely accepted as a noninvasive means of acquiring high-resolution images of the retina that can be utilized in the diagnosis and treatment of retinal diseases [11, 12]. The evolution of OCT led to OCT angiography (OCTA) that allows quantitation of capillary perfusion in the macular and optic disc areas. The high sensitivity and specificity of OCTA has enabled the measurement of retinal capillary density and whole image vessel density (VD) in ocular diseases such as glaucoma [13], optic neuritis [14, 15], and several optic neuropathies [16, 17]. With the help of OCTA technology, we recently described a reduction of retinal VD and thinning of the inner intra-retinal layers in TAO patients compared with controls [18, 19]. In TAO patients without DON, reduced VD and a thinned retinal nerve fiber layer (RNFL) were associated with the severity of the disease and the degree of visual impairment, suggesting morphological changes might precede visual dysfunction.

The degree of DON-induced visual impairment is highly variable among TAO patients, and in some, the damage is irreversible because the diagnosis occurs too late. When conducting studies of DON in a TAO study group, it is therefore extremely important to distinguish between patients with suspected DON and those with non-DON (NDON) characteristics.

The identification of suspected DON patients, i.e., those showing the earliest signs of DON development, will facilitate the exploration of the degenerative processes that result in visual impairment. The radial peripapillary capillary vessel density (RPC-VD) has recently become a promising area in the diagnosis, management, and research of optic neuropathies [20–22]. It has also been reported that peripapillary microvascular changes can precede vision field loss [23]. Therefore, the main purpose of this study was to explore the changes in blood supply to and the structure around the optic nerve at the early stage of DON in TAO patients for whom DON had not yet been diagnosed according to the clinical criteria. The second purpose of this study was to identify related factors that induce DON-associated changes in the optic nerve vessels and structure.

## Materials and methods

### Subjects

This prospective study was performed at a single center and in accordance with the tenets of the Declaration of Helsinki. This study was approved by the ethics committee of the Eye Hospital of Wenzhou Medical University. The ethics committee approved the screening, inspection, and data collection from the patients, and all patients provided written informed consent. TAO patients were recruited from the Eye Hospital of Wenzhou Medical University from April 1, 2018, to December 1, 2019. Age- and sex-matched healthy subjects were enrolled concurrently.

**Fig. 1:**
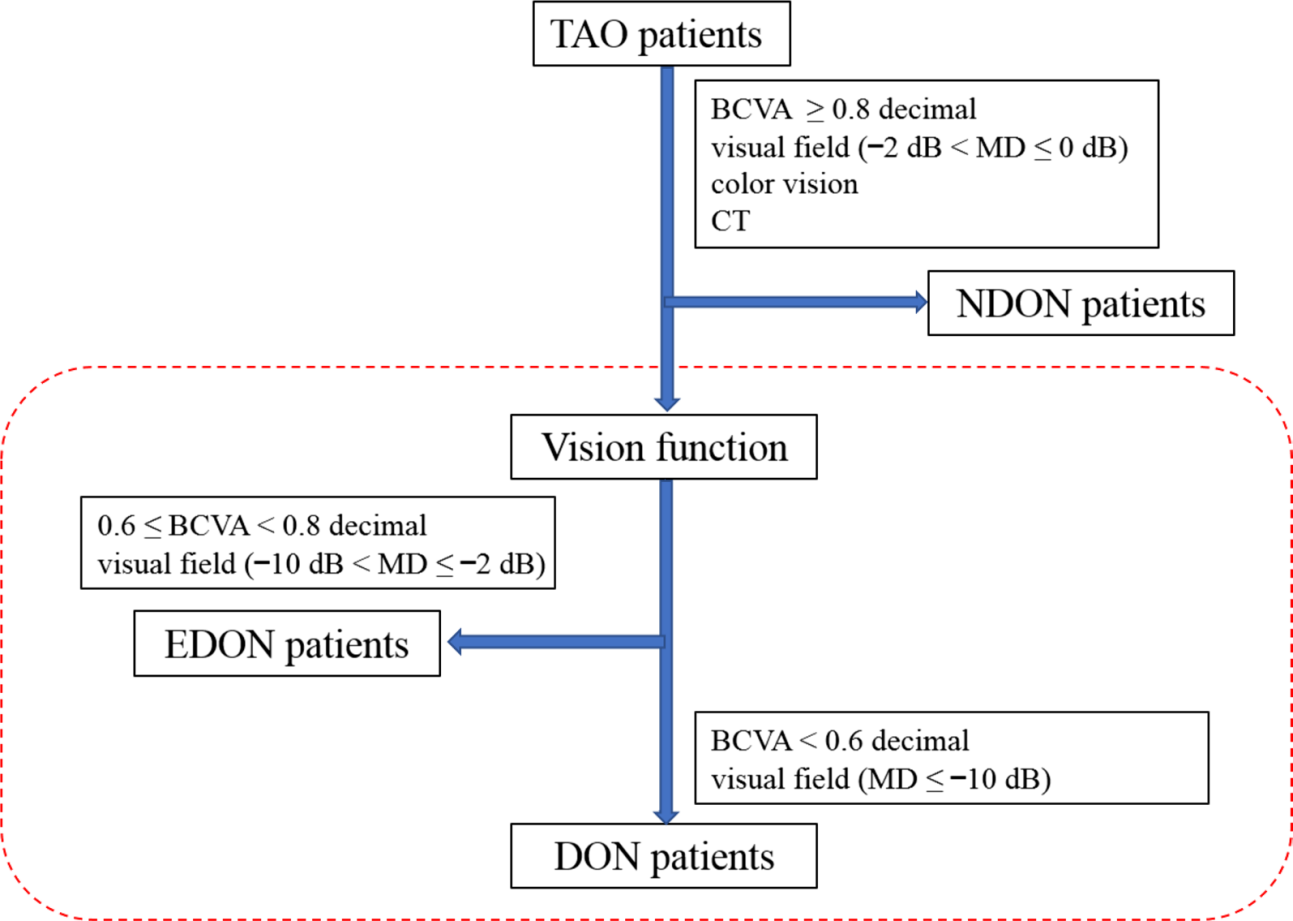
Flowchart of TAO patient grouping. TAO patients were further divided into three groups based on clinical findings. TAO, thyroid-associated ophthalmopathy; DON, dysthyroid optic neuropathy; NDON, non-DON; EDON, equivocal DON; BCVA, best-corrected visual acuity; CT, computed tomography; MD, mean deviation

TAO patients were diagnosed by an orbital disease ophthalmologist (YT) according to the Bartley international diagnostic criteria for TAO [24]. All the enrolled TAO patients in the current study were either diagnosed for the first time or those who have not receive any targeted treatment for TAO. Exclusion criteria were as follows: refractive error over 2.00 diopters (D) or under − 3.00 D of spherical equivalent (SE) or 1.50 D of astigmatism, significant media opacities, previous diagnosis of glaucoma, uveitis, or retinal disease, as well as systemic diseases like hypertension and diabetic retinopathy. Based on the clinical findings [25–27] and diagnosis, TAO patients were assigned to one of the following groups: definite DON, equivocal DON (EDON), and NDON. The TAO DON patients were diagnosed by an ophthalmologist (YT) based on a best-corrected visual acuity (BCVA) < 0.6 decimal, apparent visual field (VF) defect of ≤ − 10 dB mean deviation (MD) in Humphrey perimetry, and optic nerve head (ONH) and computerized tomography (CT) scanning results [25]. Patients placed into the NDON group had a normal BCVA (≥ 0.8 decimal), visual field (− 2 dB < MD ≤ 0 dB), color vision, and normal ONH and CT scanning results. Patients whose visual function test results were between those of DON and NDON were diagnosed as EDON (0.6 ≤ BCVA < 0.8 decimal; − 10 dB < MD ≤ − 2 dB) [25, 27]. These patients were those that had slight visual impairment but could not be diagnosed as DON (Fig. 1).

### Ophthalmic and systemic examination

All patients underwent a series of ocular examinations, including slit-lamp biomicroscopy, and measurements of refraction, BCVA, axial length (AL), intraocular pressure (IOP), exophthalmometry, visual fields, color vision, and ophthalmoscopy. Demographic information of all patients were collected, including age, sex, and duration of hyperthyroidism. Clinical laboratory measurements of biochemical indicators in sera including free triiodothyronine free thyroxine, and thyroid-stimulating hormone were performed. Control subjects underwent the same tests as the TAO patients, except for laboratory serum biochemical indicators and visual field tests. The severity of thyroid eye disease was graded according to the NOSPECS classification (N = no symptoms or signs, O = only signs, S = soft tissue involvement, P = proptosis, E = extraocular muscle involvement, C = corneal involvement, and S = sight loss due to optic nerve compression) [28]. The clinical activity scoring (CAS) system was used to establish the TAO disease activity scores for each patient [29].

### OCT imaging procedure

**Fig. 2:**
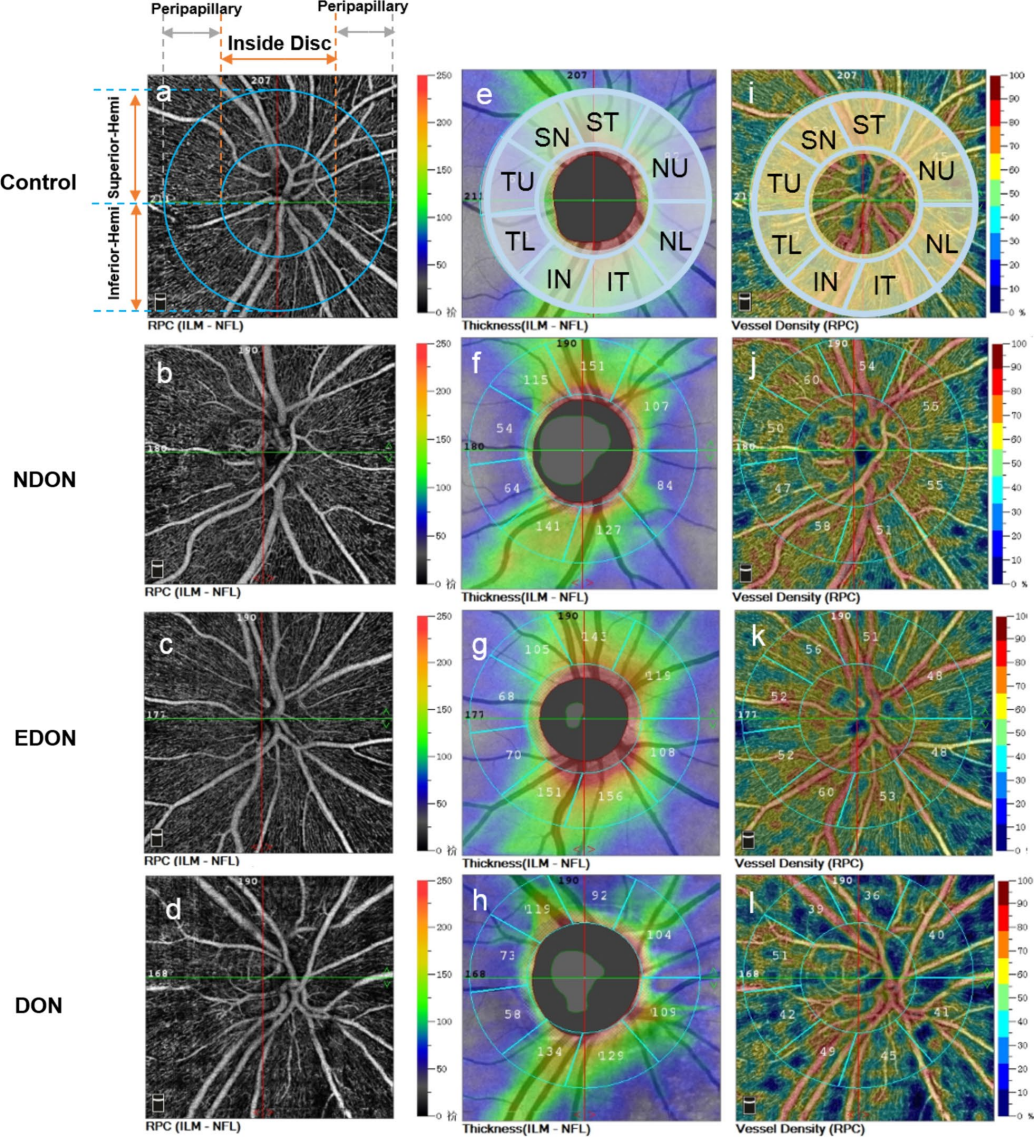
Representative OCTA images of the control, NDON, EDON, and DON groups. **a**–**d** Peripapillary vessel density maps from eyes representative of control, NDON, EDON, and DON groups. **e**–**h** P-RNFL in the Garway-Heath map sectors (radius = 1.00 mm from the optic disc boundary) **i**–**l** Circum-papillary vascular density of the RPC layer with the Garway-Heath map sectors. OCTA, optical coherence tomography angiography; DON, dysthyroid optic neuropathy; NDON, non-DON; EDON, equivocal DON; p-RNFL, peripapillary retinal nerve fiber layer; SN, superior nasal; ST, superior temporal; NU, nasal upper; NL, nasal lower; IT, inferior temporal; IN, inferior nasal; TL, temporal lower; TU, temporal upper

All enrolled subjects were imaged by the RTVue XR Avanti spectral domain OCT system (Optovue, Inc., Fremont, CA, USA) equipped with AngioVue for OCTA. Retinal blood vessels were delineated using the split-spectrum amplitude decorrelation angiography algorithm applied to the OCTA images [30]. Motion artifacts were minimized with the eye tracking system. The Angio disc OCTA scan was performed using volumetric scans covering an area of 4.5 × 4.5 mm. The peripapillary area was overlaid by annular contour lines of 2 and 4 mm in diameter around the disc margin. The software automatically fits an ellipse to the ONH margin (Fig. 2).

Peripapillary VD parameters, including the ONH whole image vessel density (ONH-wiVD) and the RPC-VD, were generated from 4.5 × 4.5 mm cube angiography scans centered on the ONH. Vascularity data were automatically converted to a VD map, where VD was calculated as the percentage area occupied by blood vessels. Based on the Garway-Heath map, the software automatically generated the VD of the global 360-degree area, the superior hemifield, and the inferior hemifield. The peripapillary region was further divided into eight sectors [temporal upper (TU), superior temporal (ST), superior nasal (SN), nasal upper (NU), nasal lower (NL), inferior nasal (IN), inferior temporal (IT), and temporal lower (TL)] based on the modified Garway-Heath map [31]. In addition to the VD indices, the OCT platform also segmented the peripapillary retinal nerve fiber layer (p-RNFL) in the ONH B-scan. All VD and retina thickness parameters were automatically calculated using the built-in program (Fig. 2). Poor image quality (defined as images with a signal quality score of less than 6), images with segmentation errors, and outstanding motion artifacts were excluded from the study.

### Statistical analysis

All continuous variables were expressed as mean ± standard deviation and analyzed with SPSS software (version 23.0; SPSS, Inc., Chicago, IL, USA). The generalized estimating equation (GEE) method was used to account for correlations between both eyes of the same patients. One-way analysis of variance (ANOVA) was used to test for differences among the control, NDON, EDON, and DON groups. Bonferroni correction was used for post hoc multiple comparisons between groups. Differences between sex within each of the four groups were determined by the χ^2^ test. Pearson correlation was performed to analyze the relationships among the visual function and the retinal microstructural parameters. The correlation between influence factors [sex, age, body-mass index (BMI), IOP, CAS, proptosis, duration of disease) and the microvascular density parameters (ONH-wiVD, RPC-VD) were tested by univariate correlation and multiple linear regression analyses.

## Results

### Patient characteristics

TAO patients (n = 63) were enrolled and assigned to the DON group (n = 19, 31 eyes), the NDON group (n = 24, 42 eyes), and the EDON group (n = 20, 30 eyes). Thirty-four control subjects (50 eyes) were also included. Only one eye was used in some of the TAO patients and control subjects due to poor coordination and subpar image quality. There were no differences in age (*P* = 0.111), sex distribution (*P* = 0.438), SE (*P* = 0.127), or AL (*P* = 0.666) among the four groups (Table 1). However, there were differences in IOP (*P* < 0.001), BCVA (*P* < 0.001), and exophthalmos (*P* < 0.001) among the groups (Table 1). Even though there were no significant differences of the duration time of hyperthyroidism between the three TAO groups (*P* = 0.122, Table 1), the visual field mean deviation (VF-MD) (P < 0.001), visual field pattern standard deviation (VF-PSD) (*P* < 0.001), and CAS score (*P* = 0.02) of the DON group were higher than the other two groups (Table 1).

**Table 1:** Demographic characteristics of all subjects

Parameters	Control n = 34	NDON n = 24	EDON n = 20	DON n = 19	*P* value
Eyes	50	42	30	31	
Age (years)	42.8 ± 11.9	41.3 ± 11.9	41.4 ± 10.1	49.2 ± 9.1	0.111*
Sex (M:F)	14:20	10:14	9:11	11:8	0.438†
SE (diopter)	− 1.06 ± 1.49	− 0.74 ± 1.18	− 1.06 ± 1.90	− 0.17 ± 1.33	0.127*
IOP (mmHg)	12.30 ± 2.21	15.47 ± 2.49	15.78 ± 3.75	20.71 ± 4.85	< 0.001*
AL (mm)	23.01 ± 3.00	23.47 ± 0.81	23.30 ± 0.81	22.98 ± 0.67	0.666*
BCVA (logMAR)	− 0.02 ± 0.04	− 0.01 ± 0.03	0.03 ± 0.14	0.51 ± 0.53	< 0.001*
Exophthalmos (mm)	15.17 ± 0.75	17.38 ± 2.50	17.70 ± 3.15	21.17 ± 2.89	< 0.001*
Duration (years)	NA	2.1 ± 3.3	6.5 ± 10.3	3.2 ± 4.9	0.122*
VF-MD (dB)	–	− 0.27 ± 1.50	− 4.36 ± 1.83	− 17.80 ± 6.34	< 0.001*
VF-PSD (dB)	–	2.20 ± 1.04	3.84 ± 2.17	8.36 ± 1.81	< 0.001*
CAS (Score)	–	1.44 ± 1.40	1.70 ± 1.94	3.43 ± 1.72	0.020*

Data are presented as mean ± standard deviation. *Control* = control eyes; *TAO* = thyroid-associated ophthalmopathy; *DON* = dysthyroid optic neuropathy; *NDON* = non-DON; *EDON* = equivocal DON; *M* = male; *F* = female; *SE* = spherical equivalent; *IOP* = intraocular pressure; *AL* = axial length; *BCVA* = best-corrected visual acuity; *VF-MD* = visual field mean deviation; *VF-PSD* = visual field pattern standard deviation; *CAS* = clinical activity scoring; *–* = not performed; *NA* = not applicable. Overall statistical significance for each variable was determined by One-way ANOVA. *P* values in bold indicate statistical significance. *One-way ANOVA. ^†^χ^2^ test

### Peripapillary vessel density parameters

Using one-way ANOVA, the ONH-wiVD of each grid section and the RPC-VD in all areas were significantly different between groups (all *P* < 0.01, Table 2). In the post hoc pairwise analysis, the VD in the ONH-wiVD of the superior-hemi sector in EDON eyes was significantly lower than those in healthy controls (*P* = 0.037, Table 2). There were no significant differences in any of the ONH-wiVD indices between the NDON and control groups (all *P* > 0.05). Compared with healthy controls, the EDON eyes had significantly lower vessel densities in the whole RPC-VD images, and in all sectors (all *P* < 0.05, Table 2). There were no significant differences in ONH-wiVD between the NDON and the EDON groups (*P* > 0.05, Table 2). However, RPC-VD in EDON group was significantly decreased in the peripapillary area (*P* < 0.05, Table 2). NDON patients had significantly reduced RPC-VD inside the optic disc area when compared to control groups (*P* = 0.023, Table 2).

**Table 2:** Comparisons of the ONH-wiVD and RPC-VD among control and TAO patients

	Regions	Control	NDON	EDON	DON	*P* value	Post hoc analysis *P* values		
							C vs. N	C vs. E	N vs. E
ONH-wiVD (%)	Whole	57.43 ± 2.68	56.86 ± 1.81	56.22 ± 3.42	53.98 ± 3.90	**< 0.001**	0.373	0.086	0.365
	Inside disc	61.70 ± 3.65	59.92 ± 4.39	60.69 ± 3.97	57.28 ± 5.17	**< 0.001**	0.058	0.324	0.454
	Peripapillary	59.74 ± 2.61	59.41 ± 2.05	58.23 ± 3.71	56.55 ± 4.69	**< 0.001**	0.645	0.054	0.133
	Superior-hemi	60.21 ± 3.05	59.59 ± 2.03	58.55 ± 3.65	56.76 ± 4.54	**< 0.001**	0.388	**0.037**	0.194
	Inferior-hemi	59.28 ± 2.43	59.18 ± 2.30	57.89 ± 3.90	56.26 ± 4.99	**0.001**	0.892	0.088	0.115
RPC-VD (%)	Whole	50.66 ± 2.50	50.14 ± 1.77	49.20 ± 3.28	47.42 ± 3.70	**< 0.001**	0.376	**0.025**	0.160
	Inside disc	52.07 ± 4.67	49.67 ± 5.03	49.40 ± 4.74	47.13 ± 5.67	**< 0.001**	**0.023**	**0.022**	0.818
	Peripapillary	53.24 ± 2.61	53.04 ± 2.53	51.38 ± 3.71	50.20 ± 4.58	**< 0.001**	0.768	**0.016**	**0.038**
	Superior-hemi	53.29 ± 2.99	53.02 ± 2.63	51.47 ± 3.62	50.29 ± 4.75	**0.001**	0.711	**0.024**	0.061
	Inferior-hemi	53.17 ± 2.57	52.75 ± 2.27	51.27 ± 4.07	50.12 ± 4.66	**< 0.001**	0.547	**0.015**	0.067

Data are presented as mean ± standard deviation for all subjects in each group. *Control or C* = control eyes; *TAO* = thyroid-associated ophthalmopathy; *DON* = dysthyroid optic neuropathy; *NDON or N* = non-DON; *EDON or E* = equivocal DON; *ONH-wiVD* = optic nerve head whole image vessel density; *RPC-VD* = radial peripapillary capillary vessel density; *–* = not performed; *NA* = not applicable. *P* values in bold indicate statistical significance

There were significant differences in the RPC-VD values in the SN, ST, TU, TL, and IN sectors among the four groups (one-way ANOVA, *P* < 0.05, Table 3). The RPC-VD in the ST, TU, and TL sectors in the EDON group were significantly lower than those in the control group (post hoc tests, all *P* < 0.05, Table 3). Compared to the NDON group, the vessel densities of the EDON group were significantly lower in the ST and TU sectors of the RPC-VD images (*P* < 0.05, Table 3). When compared to the controls, the RPC-VD of the NDON TAO patients was not significantly lower (*P* > 0.05), except for the TL sector (*P* = 0.019, Table 3).

**Table 3:** Comparisons of the RPC-VD among control and TAO patients

Regions	Control	NDON	EDON	DON	*P* value	Post hoc analysis *P* values		
						C vs. N	C vs. E	N vs. E
SN	51.58 ± 3.83	50.67 ± 3.82	49.86 ± 4.97	48.13 ± 7.04	**0.021**	0.374	0.137	0.497
ST	50.02 ± 4.30	49.62 ± 3.22	47.33 ± 6.24	45.94 ± 4.30	**0.001**	0.693	**0.017**	**0.049**
TU	49.77 ± 3.52	49.31 ± 3.24	46.50 ± 5.64	44.61 ± 5.30	**< 0.001**	0.618	**0.001**	**0.008**
TL	54.21 ± 4.52	51.88 ± 3.26	50.00 ± 5.82	51.87 ± 5.37	**0.002**	**0.019**	**< 0.001**	0.098
IT	58.08 ± 3.35	57.71 ± 3.52	56.45 ± 6.91	56.97 ± 6.42	0.500	0.728	0.162	0.291
IN	54.14 ± 5.10	53.29 ± 4.06	53.83 ± 2.61	50.53 ± 5.72	**0.006**	0.377	0.775	0.617
NL	56.64 ± 3.97	56.29 ± 3.72	56.80 ± 2.96	54.87 ± 4.11	0.160	0.666	0.853	0.574
NU	54.99 ± 5.11	55.76 ± 3.60	53.70 ± 4.31	52.77 ± 6.93	0.062	0.469	0.271	0.089

Data are presented as mean ± standard deviation for all subjects in each group. *RPC-VD* = radial peripapillary capillary vessel density; *Control or C* = control eyes; *TAO* = thyroid-associated ophthalmopathy; *DON* = dysthyroid optic neuropathy; *NDON or N* = non-DON; *EDON or E* = equivocal DON; *ST* = superior temporal; *SN* = superior nasal; *TU* = temporal upper; *TL* = temporal lower; *IT* = inferior temporal; *IN* = inferior nasal; *NL* = nasal lower; *NU* = nasal upper; *P* values in bold indicate statistical significance

### P-RNFL thickness

The differences of p-RNFL thickness among the four groups were significant in the IT (*P* = 0.033), IN (*P* = 0.009), and NL (*P* = 0.010) sectors (Table 4). Compared with the control group, NDON patients had a thinner p-RNFL thickness in the TU and NL sectors (*P* = 0.034 and 0.014, respectively) and increased thickness in the IT sector (*P* = 0.015). EDON patients had significantly thinner p-RNFLs than the control group in the TU sector (*P* = 0.027) and increased thickness in the IT sector (*P* = 0.012). There were no significant differences in p-RNFL thickness between the NDON and EDON groups (*P* > 0.05), except for the IN sector (*P* = 0.030, Table 4).

**Table 4:** Thickness comparisons of p-RNFL among control and TAO patients

Regions	Control	NDON	EDON	DON	*P* value	Post hoc analysis *P* values		
						C vs. N	C vs. E	N vs. E
SN	148.86 ± 31.04	148.02 ± 18.24	143.04 ± 27.05	141.26 ± 36.58	0.604	0.889	0.391	0.477
ST	111.16 ± 20.97	110.79 ± 13.79	111.93 ± 24.52	105.94 ± 26.67	0.669	0.933	0.876	0.823
TU	103.24 ± 20.97	94.26 ± 13.79	92.90 ± 24.52	93.19 ± 26.67	0.051	**0.034**	**0.027**	0.777
TL	155.60 ± 21.09	159.07 ± 20.11	147.17 ± 31.98	154.40 ± 39.14	0.354	0.548	0.192	0.076
IT	137.04 ± 37.52	152.79 ± 19.84	155.24 ± 28.47	147.00 ± 31.57	**0.033**	**0.015**	**0.012**	0.739
IN	75.22 ± 14.57	73.21 ± 12.23	80.43 ± 16.48	68.33 ± 11.08	**0.009**	0.487	0.103	**0.030**
NL	90.29 ± 27.98	80.39 ± 11.02	87.17 ± 11.60	77.17 ± 13.04	**0.010**	**0.014**	0.474	0.134
NU	138.46 ± 24.09	136.76 ± 30.01	141.03 ± 27.53	134.94 ± 35.80	0.861	0.780	0.702	0.539

Data are presented as mean ± standard deviation for all subjects in each group. *Control or C* = control eyes; *TAO* = thyroid-associated ophthalmopathy; *DON* = dysthyroid optic neuropathy; *NDON or N* = non-DON; *EDON or E* = equivocal DON; *p-RNFL* = peripapillary retinal nerve fiber layer; *ST* = superior temporal; *SN* = superior nasal; *TU* = temporal upper; *TL* = temporal lower; *IT* = inferior temporal; *IN* = inferior nasal; *NL* = nasal lower; *NU* = nasal upper; *P* values in bold indicate statistical significance

### Relationships among the peripapillary vessel density, p-RNFL thickness, visual function parameters, and risk factors

We analyzed the correlation of peripapillary structural and microvascular parameters with visual function parameters, including BCVA, VF-MD, and VF-PSD. The ONH-wiVD and RPC-VD were positively associated with VF-MD (r = 0.344 to 0.347), but negatively correlated with BCVA and VF-PSD (r = − 0.285 to − 0.362) (all *P* < 0.05, Table 5). The p-RNFL was positively associated with VF-MD (r = 0.255, *P* = 0.023), and negatively correlated with VF-PSD (r = − 0.247) (*P* = 0.029).

**Table 5:** Pearson correlation coefficient matrix on vessel density parameters and visual functions

Variables	p-RNFL	RPC	ONH-wiVD
BCVA (logMAR)	− 0.099 (0.351)	− 0.285 (0.006)	− 0.347 (0.001)
VF-MD	0.255 (0.023)	0.344 (0.002)	0.347 (0.002)
VF-PSD	− 0.247 (0.029)	− 0.351 (0.001)	− 0.362 (0.001)

The correlation coefficient *r* is displayed outside parentheses. The *P* value is displayed within the parentheses. *p-RNFL* = peripapillary retinal nerve fiber layer; *RPC-VD* = radial peripapillary capillary vessel density; *ONH-wiVD* = optic nerve head whole image vessel density; *BCVA* = best-corrected visual acuity; *VF-MD* = visual field mean deviation; *VF-PSD* = visual field pattern standard deviation

We also assessed the relationships between the peripapillary VD and risk factors, including sex, age, BMI, IOP, proptosis, duration of disease, and CAS. Univariate correlation analysis showed that ONH-wiVD and RPC-VD of the TAO groups were negatively correlated with only IOP (r = − 0.296, *P* = 0.006; r = − 0.258, *P* = 0.016, respectively). Multiple linear regression analyses showed that there were no risk factors, including IOP, that were significantly correlated with vascular density changes.

## Discussion

To the best of our knowledge, this is the first study to use OCTA to investigate the abnormal alterations of retinal VD in EDON patients. Here, we aimed to explore the changes of peripapillary morphology and to evaluate the correlation between morphological parameters, particularly RPC-VD, with the visual function in early DON patients. The results showed that peripapillary VD significantly differs between EDON and healthy eyes, but the mean retinal thickness of p-RNFL was not significantly changed. The visual impairment was closely related to the decrease of peripapillary capillary VD. The findings of the study will enable further understanding of the pathophysiology of DON and explore the morphological parameters of early visual function impairment.

The complete mechanism and pathogenesis underlying DON injury has not been fully elucidated, but elevated retrobulbar pressure, active intra-orbital inflammation, vascular insufficiency, and optic nerve stretching by proptosis are currently reported to play essential roles [7]. Although apical compression of the optic nerve is a major risk factor for the development and progression of DON, vascular factors, such as ONH perfusion impairment, have also emerged as important risk factors [26]. Previous studies of the patterns of vascular damage in TAO patients were predominantly based on the evaluation of large blood vessels by color Doppler imaging [10, 32–34]. Konuk et al. [10] found that blood flow velocity in the superior ophthalmic vein was lower in DON patients. They speculated that increased IOP and the compression of the orbital soft tissues in the bony orbit could imitate orbital compartment syndrome, and thus be responsible in part for these findings. In addition, they speculated that the decrease in orbital venous outflow increases the severity of TAO and may play a role in the pathology of DON.

With the recent advent of OCTA, which allows reproducible and quantitative assessments of the peripapillary and macular microvascular status, understanding of the reduced ONH and peripapillary microvasculature in DON has improved considerably. Based on OCTA imagery, Zhang et al. [26] found that the retina VD in the peripapillary area of DON patients was significantly decreased. Their results also suggested that the peripapillary VD was decreased in some areas of NDON patients. In our study, we found that both the ONH-wiVD and RPC-VD in DON patients were lower in all regions than those of the controls. However, the decrease of peripapillary VD in the NDON group was not statistically significant, which is contrary to Zhang et al.’s [26] findings probably due to the different definition of NDON that was used in that study. They defined NDON as patients with BCVA ≥ 0.6 decimal and MD > − 10 dB. However, we subdivided similar patients into the EDON (0.6 ≤ BCVA < 0.8 decimal; − 10 dB < MD ≤ − 2 dB) and NDON (BCVA ≥ 0.8 decimal, − 2 dB < MD ≤ 0 dB) groups. In our study, the RPC images, which excluded large blood vessels, of EDON eyes had significantly lower vessel densities in all areas than did the control eyes, the vessel densities of EDON eyes were also much lower in the peripapillary area than NDON eyes. In contrast, there was no significant change in ONH-wiVD. Large blood vessels are not easy to close, and they tend to remain open even in the late stage of the disease. Therefore, we speculate that the changes in the microcirculation had occurred in the early stages of DON.

We further divided the peripapillary region into eight sectors and analyzed the RPC-VD and p-RNFL thickness, which are valuable indicators in diagnosing and monitoring DON. An intriguing finding was that RPC-VD in the ST, TU, and TL sectors of the EDON group were significantly lower than those in the controls. In addition, the RPC-VD in the ST and TU sectors of the EDON group was also significantly decreased, compared to the NDON group. However, in most sectors, the p-RNFL thickness around the ONH was not significantly changed in the EDON group, indicating that the vessel densities around the ONH in the temporal and upper sectors are more likely to be affected in the early stage of DON.

Structural changes commonly occur as the disease progresses, but there has been no consistent conclusion surrounding the trend of structural change [35]. Sayin and Yeter [35] and Forte et al. [36] reported the p-RNFL thickness was significantly thinner in the TAO patients. Park et al. [37] also found that the mean temporal p-RNFL thickness was thinner in chronic DON when compared with acute DON and control eyes. Nevertheless, Meirovitch and colleagues [38] showed that the p-RNFL in eyes of TAO patients were significantly thicker in the superior, inferior, and nasal quadrants compared to the eyes of the healthy control group. Another study found that 56% of TAO patients with definite optic neuropathy had optic disk edema and concluded that it was a specific indicator of optic neuropathy [25]. Combining the insignificant difference between healthy controls and EDON groups in structural parameters in this sample, we speculate that the disk edema derived from orbit inflammation. This might be the reason for the insignificant difference of the p-RNFL thickness in the early stage of the disease.

In general, for the TAO groups, the relationship between the vascular (ONH-wiVD and RPC-VD) and functional characteristics (BCVA, VF-MD, VF-PSD) was stronger than the relationship between the structural (p-RNFL) and functional characteristics, which was consistent with a previous study in DON eyes [26]. On the one hand, this stronger relationship between vascular and functional characteristics may be because the vascular flow is reduced in eyes with damaged ganglion cells, even before obvious p-RNFL reduction. On the other hand, retinal thickness can be easily affected by retinal edema either in the optic disc area or the macular area. In a previous report, RNFL and macular ganglion cell layers were not different between DON eyes and fellow NDON eyes although VF sensitivity was substantially worse in the DON eyes [18, 26].

Our study is also the first to detect the microvascular characteristics of the TAO patients with EDON, who have normal visual functions, ONH structure and organization, and CT scanning results. Thus, the vascular indicators are more significant than structural indicators, which is important to know during early screening of patients suspected of having DON.

Among the risk factors, the decrease of VD in both ONH-wiVD and RPC-VD may be related to the increase of IOP in TAO patients, especially in the DON group where the IOP was significantly higher than in the other three groups. The IOP increase in the DON group might have been caused by the relative increase of intra-orbital contents that is characteristic of that group [35]. Some studies have shown that the increased IOP was correlated with a decrease in orbital venous outflow [10] and p-RNFL [35, 37]. After correcting for the influence of the related effects among the various risk factors, there was no obvious correlation with the decrease of the peripapillary VD. This indicates that the decline of retinal VD may be an independent risk factor in the early stage of TAO and should be further studied with a longitudinal design.

There are three limitations of this study. First, the number of enrolled eyes was relatively small, and there were relatively few cases of DON at the time of OCTA imaging. The small sample size could have affected the reliability of the p-RNFL and RPC-VD measurements, which were variable among the patients, even after we excluded the images of poor quality. In future studies, we will combine the characteristic parameters of the macular and optic disc areas, hoping to make possible the early diagnosis of patients with suspected DON. Third, we did not distinguish between the acute active phase and the chronic phase of DON. The main subjects of this study were the patients without obvious DON, whose CAS score did not increase, so the inflammatory component is not a likely confounder in the analysis of our results. In the future, we will report in more depth on the EDON group with longitudinal studies to monitor the development of the retinal structural and microvascular damage during the transition from chronic phase to active phase.

## Conclusions

In conclusion, the findings of this study suggest that the ONH VD, including RPC-VD and ONH-wiVD, were involved in TAO, probably as early as the detection of visual function irregularities in the clinic. The temporal and upper region vessel densities were more likely to be affected in the early stage of the disease. Our study also revealed the relationship between the visual function and morphological changes of the retinal structure and microvasculature around the disc. The combined use of spectral domain OCT and OCTA technologies offer a new method for early diagnosis of the suspected DON patients, and the prediction of progression for these early DON stage to more advanced stages.

## Data Availability

All relevant data are in the manuscript together with its supporting files.
